# Skin perfusion and oxygen saturation after mastectomy and radiation therapy in breast cancer patients

**DOI:** 10.1016/j.breast.2024.103704

**Published:** 2024-03-06

**Authors:** Sherif Elawa, Ingemar Fredriksson, Ingrid Steinvall, Johan Zötterman, Simon Farnebo, Erik Tesselaar

**Affiliations:** a Department of Biomedical and Clinical Sciences, Linköping University, Linköping, Sweden; b Department of Medical Radiation Physics, Department of Health, Medicine and Caring Sciences, Linköping University, Linköping, Sweden; c Department of Plastic Surgery, Hand Surgery, and Burns, Linköping University, Linköping, Sweden; d Department of Biomedical Engineering, Linköping University, Linköping, Sweden; e Perimed AB, Järfälla, Stockholm, Sweden

## Abstract

The pathophysiological mechanism behind complications associated with postmastectomy radiotherapy (PMRT) and subsequent implant-based breast reconstruction are not completely understood. The aim of this study was to examine if there is a relationship between PMRT and microvascular perfusion and saturation in the skin after mastectomy and assess if there is impaired responsiveness to a topically applied vasodilator (Methyl nicotinate – MN).

Skin microvascular perfusion and oxygenation >2 years after PMRT were measured using white light diffuse reflectance spectroscopy (DRS) and laser Doppler flowmetry (LDF) in the irradiated chest wall of 31 women with the contralateral breast as a control. In the non-irradiated breast, the perfusion after application of MN (median 0.84, 25th–75th centile 0.59–1.02 % RBC × mm/s) was higher compared to the irradiated chest wall (median 0.51, 25th–75th centile 0.21–0.68 % RBC × mm/s, p < 0.001). The same phenomenon was noted for saturation (median 91 %, 25th–75th centile 89–94 % compared to 89 % 25th–75th centile 77–93 %, p = 0.001). Eight of the women (26%) had a ≥10 % difference in skin oxygenation between the non-irradiated breast and the irradiated chest wall.

These results indicate that late microvascular changes caused by radiotherapy of the chest wall significantly affect skin perfusion and oxygenation.

## Highlights

• Skin perfusion and oxygenation in the breast are affected several years after radiotherapy.
• Complications following reconstructive surgery after PMRT may, at least partly, be explained by changes in microvascular blood flow or microvascular responsiveness.
• MN provocation followed by white light diffuse reflectance spectroscopy (DRS) combined with Laser Doppler Flowmetry (LDF) may be a valuable clinical tool prior to deciding surgical procedures after PMRT.

## 1 Introduction

Radiotherapy is an important component in treating and preventing recurrence of advanced breast cancer [[1], [2], [3]]. Up to 40 percent of women with breast cancer undergo a total mastectomy [4,5]. For patients who undergo total mastectomy, there is evidence that postmastectomy radiotherapy (PMRT) is beneficial when there is a high risk of recurrence (four or more positive lymph nodes, tumor of ≥5 cm, or invading skin or musculature) [[6], [7], [8], [9], [10], [11]].

Many patients desire to proceed with a breast reconstruction after the cancer is removed, with the aim to recreate shape and symmetry of the breast. Implant based reconstruction techniques are the most used but have been shown to be more prone to complications when an adjuvant radiotherapy protocol is used [[12], [13], [14]]. The most common post radiation complications in patients with total mastectomy and implant reconstruction include infections and tissue morbidity (fat necrosis, skin closure problems) in the short term, and capsular contracture in the long-term. Autologous reconstruction using perforator-based tissue flaps (for example the deep inferior epigastric perforator (DIEP), superficial inferior epigastric artery (SIEA) and transverse upper gracilis (TUG) flaps) and microsurgical techniques can be used as an alternative to implant-based reconstruction, to reduce these risks. Long-term patient-reported outcomes, including health related quality of life, have been shown to be significantly better following autologous reconstruction compared to implant-based reconstruction and the frequency of post-reconstruction complications has been lower [15]. Autologous reconstruction, however, faces several barriers including the fact that it is a considerably more technically complicated surgery and that it is relatively less reimbursed. Taking all factors into account, including co-morbidity, it is challenging to decide which reconstructive alternative will be best suitable for each patient. The superiority of using autologous reconstruction is believed to be due to the transfer of vascularized tissue to the reconstructed site and that autologous tissue will behave similar to a natural breast, developing ptosis over time and changing with the weight of the patient. By contrast, women with unilateral implant-based reconstruction, will typically develop an asymmetry that will become more marked over time. Other complications such as capsular contracture are more likely to develop, requiring further interventions to maintain an acceptable cosmetic results. However, according to this MROC study [16] 90% of patients with irradiated implants reported being satisfied, of which 94% of patients would choose the same method of reconstruction again.

The pathophysiological mechanisms behind PMRT complications are not entirely understood but are likely to be attributed to tissue fibrosis within the radiated tissue. It has been speculated that the fibrosis formation is related to relatively late changes in the microcirculation of the skin (4–12 months). However, it seems like the initial radiotherapy related alterations in microvascular blood flow and microvascular responsiveness [17] are reversed over time and that other factors, including reduced tissue oxygenation related to radiation exposure, may cause this effect.

The relationship between fibrosis and tissue hypoxia is however debated, as oxygen is crucial for adequate radiation response, and hypoxia is postulated to be a major source of radiation resistance [18]. Therefore, measuring tissue oxygenation may be a way to identify radiation-induced fibrosis that could potentially lead to complications in subsequent reconstructive surgery.

The aim of this study was to examine if there is a relationship between PMRT and microvascular perfusion and saturation in the skin after mastectomy and assess if there is impaired capillary capacity measured as the responsiveness to a topically applied vasodilator (Methyl nicotinate – MN).

## 2 Methods

Thirty-one women referred to a university reconstructive plastic surgery department for breast reconstruction after unilateral mastectomy and PMRT more than two years prior were enrolled in the study. The number of participants was based on a sample size that allows for detecting a 10% change in red blood cell (RBC) oxygen saturation, accounting for a 10% loss to follow up. Before inclusion, the participants gave written informed consent. None of the patients used nicotine. Measurements were made at a room temperature of 21.0 ± 1.0 °C with the participants in a supine position. The study was carried out according to the Declaration of Helsinki and was approved by the Regional Ethics Review Board in Linköping, Dnr 2014/299-31.

### 2.1 Equipment

Microcirculatory measurements were performed using a multimodal optical instrument (PeriFlux 6000 EPOS, Perimed AB, Stockholm, Sweden) [19] that integrates white light diffuse reflectance spectroscopy (DRS) and Laser Doppler Flowmetry (LDF) for comprehensive microcirculatory assessment. The EPOS system acquires DRS and LDF data noninvasively using a fiberoptic probe placed in contact with the skin. The acquired data are analysed by the EPOS system using an adaptive model-based algorithm that automatically accounts for intra- and inter-individual variations in the optical and geometrical properties of the skin. This allows for a quantitative and simultaneous estimation of the skin microcirculatory oxygen saturation (S_O2_), speed resolved perfusion (perf_tot_, perf_0–1_, perf_1–10_, and perf_>10_, respectively), and (RBC) tissue fraction (C_RBC_) in real-time [19,20]. The method has been validated using simulations of light transport in realistic multilayer geometries where tissue model properties such as epidermal thickness, the number and diameter of individual vessels, C_RBC_, flow speeds, and S_O2_ were varied [[19], [20], [21]].

### 2.2 Experimental procedure

Before the experiments, the skin was cleaned using chlorhexidine ethanol (5 mg/ml, Fresenius AB, Uppsala, Sweden). Thereafter, 50 μl 20 mM of methyl nicotinate (MN, Sigma-Aldrich, Merck Group) was applied on a circle with a diameter of 3 cm of the skin under the areolar region and mastectomy scar, using a pipette [22]. We have previously shown that there was no significant difference in skin perfusion between measurement sites above or below the areolar complex/mastectomy scar [17].

**Fig. 1:**
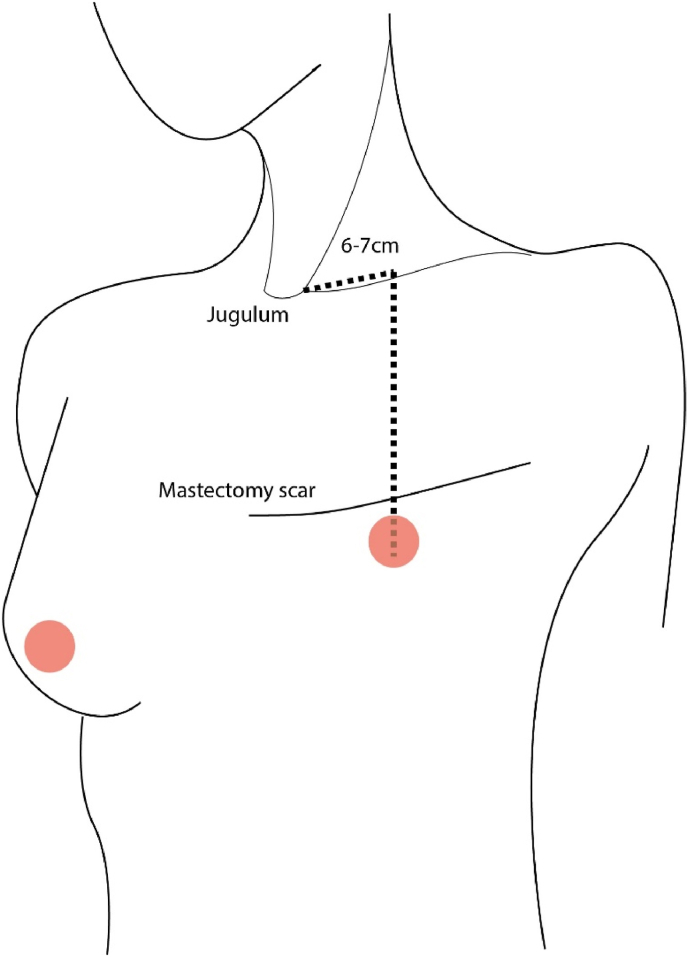
Illustration of the measurement sites. Skin perfusion and oxygen saturation were measured before and after application of methyl nicotinate on the breast below the areolar complex or the chest wall mastectomy scar.

Then, the MN was gently rubbed into the skin by hand while wearing latex gloves. Baseline perfusion was measured for 20 s on each site before applying the drug. After 15 min of MN application, perfusion was again measured for 20 s (Fig. 1).

### 2.3 Data analysis

Baseline values and responses were calculated for each subject as the median of the 20 s measurements. As the data was not normally distributed Wilcoxon signed rank tests were used to analyse two matched samples and Mann-Whitney U tests were used for unpaired data to test for differences between groups. Spearman correlation coefficients were used to describe the association between variables.

## 3 Results

Demographic data are shown in Table 1.

**Table 1:** Descriptive data on the study participants (n = 31). Data are presented as median SD or n (%).

**Number of women included**	**31**
**Physiological parameters**	***median (SD)***
Age (years)	49.6 (9.3)
Length (m)	1.65 (0.06)
Weight (kg)	70 (9.8)
BMI, kg/m	24.8 (2.6)
**Comorbidities**	**n (%)**
Diabetes Mellitus Type 2	0
Hypertension	6 (19)
**Chemotherapy**	**n (%)**
Neoadjuvant	16 (52)
Adjuvant	21 (68)
Postoperative	23 (74)
**Radiotherapy**	***median (SD)***
Total dose (Gy)	40 (5.4)
Time since last fraction (mo)	44 (37)

There was no significant difference in baseline skin perfusion between irradiated chest wall and non-irradiated breast. RBC oxygen saturation during baseline was however significantly higher in the irradiated chest wall compared to the non-irradiated breast (58 % (48–68) vs 48 % (32–61), respectively p = 0.01) (Table 2).

**Table 2:** Saturation and perfusion values at rest and after provocation with methyl nicotinate (MN) in the irradiated chest wall and non-irradiated breast.

	Irradiated chest wall	Non-irradiated breast	p-value^a^
**Baseline, at rest**			
Saturation (%)	58 (48–68)	48 (32–61)	0.01
Perfusion (%RBC x mm)	0.13 (0.08–0.18)	0.13 (0.09–0.18)	0.51
**Provocation, MN**			
Saturation (%)	89 (77–93)	91 (89–94)	0.001
Diff >10 %	70 (67–77)	92 (89–93)	0.01
Diff <10 %	92 (87–94)	91 (89–94)	0.11
Perfusion (%RBC x mm)	0.51 (0.21–0.68)	0.84 (0.59–1.02)	<0.001

MN = Methyl nicotinate. Data are presented as median (25th–75th centile). N = 31.

a Calculated using Wilcoxon Matched Pairs Test.

**Fig. 2:**
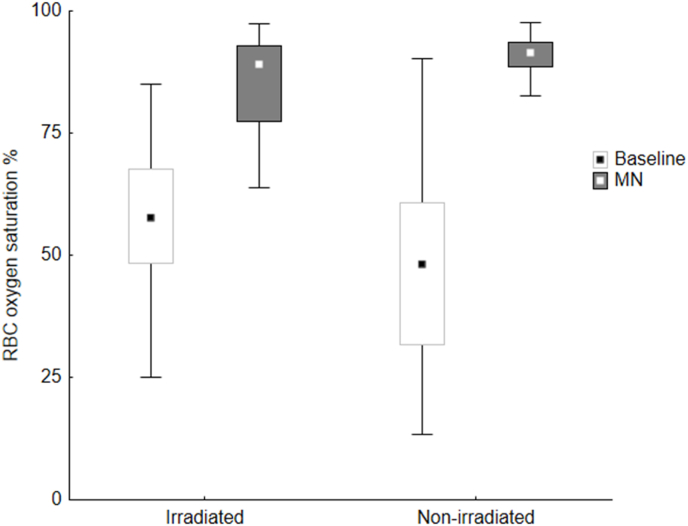
Oxygenation (%) in irradiated and non-irradiated skin before and after application of methyl nicotinate (MN). Squares indicate median, boxes indicate 25th – 75th percentile, whiskers show non-outlier range.

**Fig. 3:**
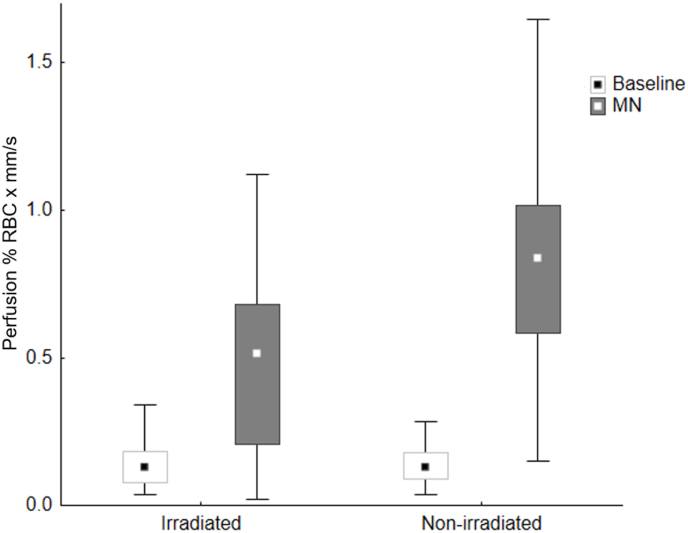
Perfusion (% RBC × mm/s) in irradiated and non-irradiated skin before and after MN Squares indicate median, boxes indicate 25th – 75th percentile, whiskers show non-outlier range.

After MN was applied, a significant increase in skin perfusion and RBC oxygen saturation was noted in both the irradiated chest wall and non-irradiated breast. In the non-irradiated breast, the perfusion after application of MN was significantly higher compared to the irradiated chest wall (0.84 (0.59–1.02) % RBC × mm/s vs 0.51 (0.21–0.68) % RBC × mm/s, respectively; p < 0.001). The same phenomenon was noted for saturation (91 % (89–94) vs 89 % (77–93), respectively; p = 0.001) (Table 2). While 81 % (25/31) of the participants had ≥10 % difference in skin perfusion between the non-irradiated breast and the irradiated chest wall, only 26 % (8/31) had a ≥10 % difference in skin oxygenation between the non-irradiated breast and the irradiated chest wall (Fig. 5). Fig. 2, Fig. 3 show oxygenation and perfusion in irradiated and non-irradiated skin before and after application of MN.

**Fig. 4:**
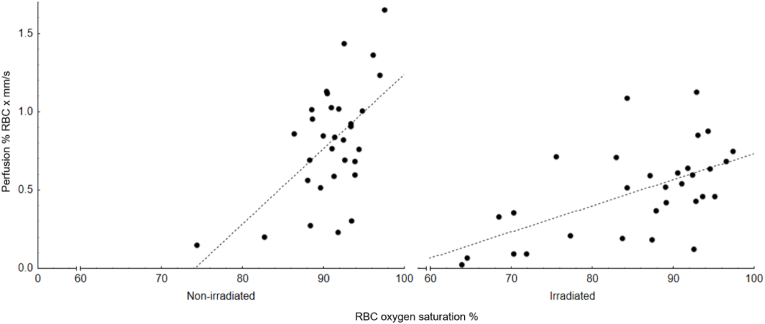
*The association between saturation and perfusion in the non-irradiated (left) and the irradiated chest wall (right) after provocation with methyl nicotinate. The correlation coefficient ρ was 0.40 (p* = *0.02)* in the non-irradiated breast and *0.58 (p < 0.001) in the irradiated chest wall.*

Correlation analysis showed that the association between skin perfusion and oxygenation after application of MN was stronger in the irradiated chest wall (ρ = 0.58; p < 0.001) than in the non-irradiated breast (ρ = 0.40; p = 0.02), which can partly be explained by the larger variation in skin perfusion in the irradiated chest wall (Fig. 4).

**Fig. 5:**
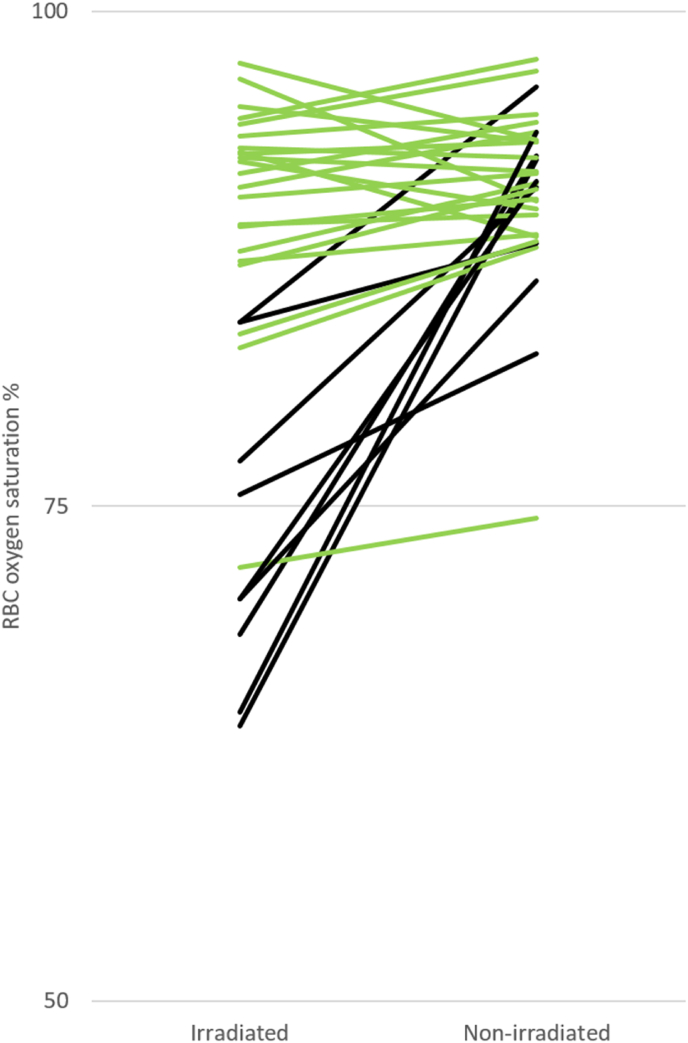
Difference in RBC oxygen saturation between irradiated chest wall and non-irradiated breasts. Black lines (26 % of patients) show patients with a >10 % difference in RBC oxygen saturation between irradiated chest wall and non-irradiated breast. Green lines (74 % of patients) show a <10 % difference in RBC oxygen saturation between irradiated chest wall and non-irradiated breast.

## 4 Discussion

The main finding in this study is that skin perfusion and oxygenation after stimulation with MN were significantly decreased in the irradiated chest wall after PMRT compared to the non-irradiated breast in patients with complete unilateral mastectomy. This finding could represent one important puzzle piece in understanding the mechanism behind tissue morbidity after surgical procedures to irradiated skin.

To our knowledge, this is the first study investigating the relationship between oxygenation and perfusion when the reactivity of skin microvasculature is challenged by a strong vasodilator.

At rest, a minor difference in oxygenation was observed, characterized by higher oxygenation levels in irradiated chest wall compared to the non-irradiated side. This indicates the subtlety and complexity of skin evaluation under resting conditions. However, following the application of MN, there was a significant reduction in oxygenation observed in irradiated chest wall as opposed to the non-irradiated breasts.

The use of topical application of MN, which initiates a prostaglandin mediated vasodilation, to increase basal perfusion in the skin has been evaluated with laser speckle contrast imaging (LSCI) [22,23]. Our results indicate that MN may have a role in identifying patients that have a decreased vascular reactivity secondary to tissue fibrosis formation after radiotherapy.

Radiation-induced fibrosis (RIF) is a long-term side effect of external beam radiation therapy for the treatment of cancer, for which radiation and the volume of tissue irradiated are the most significant risk factors. RIF usually occurs 4–12 months after radiation therapy and progresses over several years. Irradiated skin shows a reduction of thickness in the dermis and subcutaneous layer, less prominent rete ridges, and a thicker stratum spinosum layer [24].

The mechanism of RIF is similar to that of any chronic wound healing process. An initial injury incites an acute response that leads to inflammation, followed by fibroblast recruitment and activation with extracellular matrix deposition [25]. Radiation injury results from two primary mechanisms: direct DNA damage and the generation of reactive oxygen species, the last of which accounts for 60–70 % of the total damage [[26], [27], [28]]. When ionizing radiation interacts with water molecules, it forms free radicals that damages all components of cells [28]. Injured cells release chemoattractant molecules that trigger nonspecific inflammation [[29], [30], [31]]. Among the chemokines and cytokines that are released due to this inflammatory process TGF-β, is important and heavily implicated in RIF. TGF-β is responsible for a number of functions that contribute to the production of myofibroblasts. Although myofibroblasts promote endothelial cell proliferation and angiogenesis through the secretion of basic fibroblast growth factor (bFGF) [32], excess collagen reduces vascularity over time [33]. This makes fibrotic areas susceptible gradual ischemia [29,[34], [35], [36], [37]]..

In a previous study [17] in partial mastectomy patients, we used LSCI to measure skin perfusion in non-irradiated and irradiated breasts, before, directly after and up to 6 months after PMRT. No significant changes were observed in skin perfusion and microvascular responsiveness after PMRT in that study at 6 months, but oxygenation was not measured. Contrary to in that study we found a decreased perfusion after MN provocation in the current study. There are however two major differences between the two study groups. In the current study all participants had undergone a total mastectomy with RT (radiotherapy), compared to partial mastectomy and RT in the previous study. Also, the follow-up time was at least 2 years in this study, compared to 6 months in the previous study. Besides the time aspect, the amount of remaining subcutaneous tissue could have affected skin microcirculation measured as total perfusion. This may explain the differences in skin perfusion between our previous findings and the results in this study.

The skin is a radiosensitive organ, suggesting it is well-oxygenated in the resting state. Rudolph et al. described that human skin, even many decades after radiation therapy, retains normal tissue oxygenation [36]. Several studies have shown similar results [38,39]. Their conclusion was that post-radiation scarring, poor healing, and rare ulceration cannot solely be explained by ischemia, but rather be caused by other radiation effects, such as permanent changes in breast tissue fibroblasts. Our results show that skin perfusion and oxygenation decrease after radiation therapy.

In a majority of patients in our study group, skin perfusion was lower in the irradiated region compared to the non – irradiated. Our correlation analysis furthermore displays a relationship between skin perfusion and oxygenation. Reduced skin perfusion does not entirely explain the decreased oxygenation, suggesting that other mechanisms, such as radiation-induced fibrosis, may affect the saturation.

Furthermore, there is histological evidence of decreased microvascular network density and alterations to the morphology of blood vessels after radiation therapy [40]. Acutely following radiation exposure, the vessels of mice shows decreased blood supply to the tissue and lower oxygen tension, which further stimulates fibrosis [41].

Individualized selection of a reconstructive technique for each patient is a predominant factor in achieving a reconstructive success. Choosing the right technique can however be challenging.

Implant-based breast reconstruction (IBBR) is still the most performed restorative technique following mastectomy. In the US, approximately 80% of patients seeking breast reconstruction are subjected to IBBR, in contrast to 18% to autologous reconstruction (Surgeons ASoP (2020) plastic surgery statistics report 2020). According to a review by Awadeen et al. [42] irradiated breasts were 1.89 times more vulnerable to losing implants, 4.17 times more vulnerable to developing capsular contracture and 1.49 times more susceptible to develop wound infection compared to the non-irradiated breast [42].

Based on the saturation patterns seen in our study (Fig. 4), it may be possible to identify which patients that are more prone to postsurgical morbidity than others.

In our material we identified two groups of patients in regard to their ability to respond adequately to a microvascular provocation: a group that is not affected by PMRT when measuring RBC oxygen saturation and a group with a 10 % or more decline in saturation after radiation therapy. The majority of the patients (87.1 %) had a decreased perfusion after PMRT in the irradiated breast compared to the non-irradiated breast. Our hypothesis is that the group with a 10 % or more decline in saturation could have an increased risk for complications following IBBR. A key determinant of the process of healing is tissue oxygenation. Blood vessels function as conduits for the delivery of oxygen. The underlying factors that promote abnormal healing and fibrosis are still poorly understood but may be explained by chronic tissue hypoxia. In recent years, new improved methods to assess microcirculation has been developed [19,20]. With the ability to measure microcirculatory perfusion and oxygen saturation in absolute units, one of the main limitations of microcirculatory assessments has been overcome [43]. LDF and Laser Speckle contrast imaging (LSCI) are often used in studies of the microcirculation. They are based on the same principle and seemingly reflect the velocity and concentration of erythrocytes in a given volume. Previous studies in adults have reported that LSCI is far more reliable than LDF at rest [43,44]. Hodges et al. compared microvascular reactivity assessed by laser-Doppler flowmetry (LDF) and laser speckle contrast imaging (LSCI) at rest, post-occlusive reactive hyperaemia (PORH), and cycling exercise. LSCI values were higher for all groups compared to the LDF, with larger disparity as blood flow increased [45].

With our current method we combine diffuse reflectance spectroscopy (DRS) and laser Doppler Flowmetry (LDF). This method has been used to study the link between cardiovascular disease and impaired microvascular function [46]. With stimulation of MN, this novel fiberoptic technique can be a helpful tool in deciding which reconstructive technique is most suitable for the patient.

It is important to note that this study has limitations. The assessment of microvascular changes was restricted to assessment of the skin alone. Effects on subcutaneous tissues cannot be assessed using this technique. We only measured on one measurement point on each breast. The study solely relied on LDF and DRS (LDF) as the measurement tool for assessing microvascular perfusion and skin oxygenation. While this is a valuable technique, the use of additional complementary methods could have provided a more comprehensive understanding. Laser Speckle contrast imaging (LSCI) could for instance examine a larger area that can be sampled and assessed, thus reducing the known issues of vascular heterogeneity. Only one measurement was done at a routine clinical consultation prior to deciding the surgical procedure. No repeated measurements were done, which may be regarded as a limitation as the late effects of PMRT may have a time dependent impact on the microcirculation. We have, however in a previous study [17] shown that the skin perfusion was normalised already after two months. We therefore believe that the time effect in our study group is of less importance. Finally, the small sample size in our study limited our ability to investigate other potential factors. Also, all included patients were subsequently reconstructed with an autologous reconstruction where the potential impact of an impaired microvascular bed cannot be substantiated in postoperative complications.

To conclude, our findings suggest that the microvascular changes caused by radiotherapy of the breast significantly affect skin perfusion and oxygenation. The increased risk for complications during reconstructive surgery can therefore, at least partly, be explained by changes in microvascular blood flow or microvascular responsiveness.

## Funding

This research did not receive any specific grant from funding agencies in the public, commercial, or not-for-profit sectors.

## Ethical approval

Approved by the Regional Ethics Review Board in Linköping, Dnr 2014/299-31.

## CRediT authorship contribution statement

**Sherif Elawa:** Writing – original draft, Visualization, Validation, Software, Resources, Project administration, Methodology, Investigation, Funding acquisition, Formal analysis, Data curation, Conceptualization. **Ingemar Fredriksson:** Writing – review & editing, Software, Methodology, Formal analysis. **Ingrid Steinvall:** Writing – review & editing, Software, Methodology, Formal analysis. **Johan Zötterman:** Writing – review & editing, Software, Methodology, Formal analysis. **Simon Farnebo:** Writing – review & editing, Supervision, Project administration, Methodology, Funding acquisition, Formal analysis, Conceptualization. **Erik Tesselaar:** Writing – review & editing, Supervision, Software, Project administration, Methodology, Funding acquisition, Formal analysis, Conceptualization.

## Declaration of competing interest

Fredriksson is part-time employed by Perimed, AB, which is developing products related to research described in this publication. None of the other authors has disclosable conflicts of interests.
